# GP96: safeguarding Treg

**DOI:** 10.18632/oncotarget.4582

**Published:** 2015-06-22

**Authors:** Yongliang Zhang, Ephraim Ansa-Addo, Zihai Li

**Affiliations:** Department of Microbiology & Immunology, Hollings Cancer Center, Medical University of South Carolina, Charleston, SC, USA

**Keywords:** immunology and microbiology section, immune response, immunity

FOXP3 positive regulatory T cells (Tregs) are key players in maintaining peripheral immune tolerance. Multiple molecules and signaling pathways are involved in regulating Treg function. Understanding these mechanisms is vital to designing Treg-targeted immunotherapeutic strategies for cancer and other diseases. A recent study uncovered that endoplasmic reticulum resident chaperone, gp96, is essential for the function of Tregs. Genetic depletion of gp96 in Treg lineage leads to uncontrolled fatal inflammation. Mechanistically, gp96 safeguards Treg suppressive function by maintaining FOXP3 stability and regulating cell surface TGF-β bioavailability [1].

Tregs express a master transcription factor FOXP3 that controls their lineage stability and function. Although controversial, recent studies have suggested that Tregs may retain lineage plasticity, which is, the ability to switch their cell fate to various effector T cell types under circumstances such as inflammation [2]. gp96 is an essential chaperone for Toll-like receptors (TLRs) and integrins. When *hsp90b1* (encoding gp96) was deleted conditionally using FOXP3-driven cre, FOXP3 expression decreased significantly [1]. Using a cell transfer model [2], it was found that highly purified (>99%) gp96-null Tregs dynamically lose FOXP3 expression and convert to IFN-γ producing T effector cells in the lymphopenic environment, despite the fact that Treg-specific demethylated region (TSDR) in the FOXP3 promoter was persistently demethylated [1].

**Figure 1:**
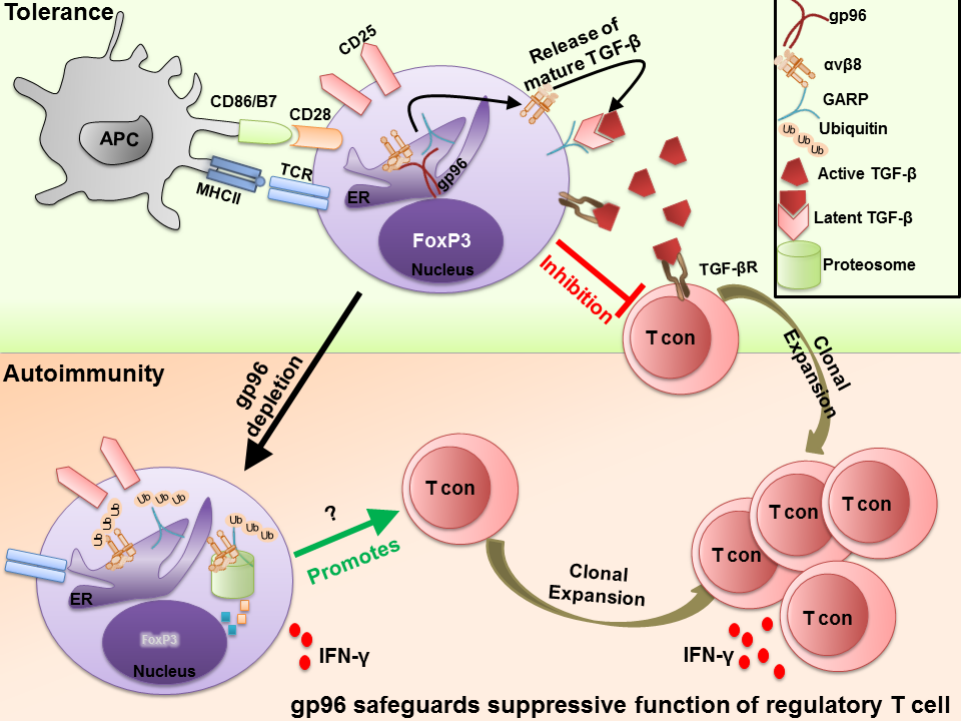
Molecular chaperone gp96 safeguards the suppressive function of regulatory T cells TGF-β exerts pleotropic functions in Treg cell biology including lineage stability, cell proliferation and suppressive function. Upon TCR stimulation, activated Treg cells express membrane latent TGF-β (mLTGF-β) on the cell surface via binding to the surface docking protein, GARP. To be functional, mLTGF-β has to be cleaved and released in an active form by integrins including αvβ8, also expressed by Treg cells. However, upon deletion of gp96 in Treg cells, TGF-β bioavailability is disrupted as the surface expression of both GARP and integrin, αvβ8, is dependent on gp96. In addition, Treg cells lose FoxP3 and convert to IFN-γ producing T cells in absence of gp96. In this setting, immune tolerance is not preserved and Treg-specific gp96 KO mice develop autoimmune disease progressively.

Further studies unveiled that gp96 unexpectedly regulates TGF-β bioavailability on the cell surface. Three forms of TGF-β have been identified: soluble and active TGF-β, latent TGF-β associated with latent TGF-β binding protein (LTBP) and the membrane latent form of TGF-β (mLTGF-β). In Tregs, mLTGF-β is highly expressed and associated to the presence of mLTGF-β docking protein, GARP (also known as LRRC32) [3]. Intriguingly, GARP shows common structural similarities with TLRs and GPIb, two proven client proteins of gp96. It was found that gp96 is obligatory chaperone for GARP. Without gp96, both GARP and mLTGF-β are completely disrupted. In addition, as expected, KO Treg cells also exhibit defective expression of multiple integrins including αV and β2 which are known to covert latent TGF-β to biologically active one. Interestingly, a recent report showed that integrin αvβ8 mediates TGF-β activation by effector Tregs and is essential for the suppression of T cell-mediated inflammation [4]. Thus, gp96 appears to play critical roles in maintaining TGF-β bioavailability and Treg cell function by chaperoning both GARP and integrins (FIGURE 1).

TGF-β exerts pleotropic functions in Treg cell biology. Treg-produced TGF-β1 controls Treg proliferation in an autocrine manner [5]. When TGF-β1 is depleted in Tregs, their populations expanded significantly. GARP-null Tregs also expand [6], as in the case of gp96 deleted Tregs [1]. Since TGF-β is a key cytokine for maintaining FOXP3 expression and suppressive function in Treg cells [7], it is thus not surprising to see the compromised Treg lineage stability due to loss of gp96-GARP-mLTGF-β axis.

The dual roles of gp96 in controlling both surface availability and activation of TGF-β may have important therapeutic implications against a variety of diseases. One such promising implication is cancer immunotherapy, as Tregs are one of the major barriers impeding anti-tumor immune responses. Dampening Treg suppression by blocking gp96 would reverse immune tolerance and thus promote cancer immunity. The challenge is how to design gp96 inhibitors that can preferentially target Tregs, a goal that is being actively pursued.
